# Rapid and sensitive detection of *Salmonella* species targeting the *hilA* gene using a loop-mediated isothermal amplification assay

**DOI:** 10.5808/gi.21048

**Published:** 2021-09-30

**Authors:** Jiyon Chu, Juyoun Shin, Shinseok Kang, Sun Shin, Yeun-Jun Chung

**Affiliations:** 1 Department of Biomedicine & Health Sciences, Graduate School, The Catholic University of Korea, Seoul 06591, Korea; 2 Department of Microbiology, College of Medicine, The Catholic University of Korea, Seoul 06591, Korea; 3 Precision Medicine Research Center, College of Medicine, The Catholic University of Korea, Seoul 06591, Korea; 4 Integrated Research Center for Genome Polymorphism, College of Medicine, The Catholic University of Korea, College of Medicine, Seoul 06591, Korea; 5 ConnectaGen, Hanam 12918, Korea; 6 Chungbuk Veterinary Services Laboratory, Chungju 27336, Korea

**Keywords:** *hilA* gene, loop-mediated isothermal amplification, point-of-care testin, *Salmonella*

## Abstract

*Salmonella* species are among the major pathogens that cause foodborne illness outbreaks. In this study, we aimed to develop a loop-mediated isothermal amplification (LAMP) assay for the rapid and sensitive detection of *Salmonella* species. We designed LAMP primers targeting the *hilA* gene as a universal marker of *Salmonella* species. A total of seven *Salmonella* species strains and 11 non-*Salmonella* pathogen strains from eight different genera were used in this study. All *Salmonella* strains showed positive amplification signals with the *Salmonella* LAMP assay; however, there was no non-specific amplification signal for the non-*Salmonella* strains. The detection limit was 100 femtograms (20 copies per reaction), which was ~1,000 times more sensitive than the detection limits of the conventional polymerase chain reaction (PCR) assay (100 pg). The reaction time for a positive amplification signal was less than 20 minutes, which was less than one-third the time taken while using conventional PCR. In conclusion, our *Salmonella* LAMP assay accurately detected *Salmonella* species with a higher degree of sensitivity and greater rapidity than the conventional PCR assay, and it may be suitable for point-of-care testing in the field.

## Introduction

*Salmonella* species are among the major pathogens that cause foodborne infections. Indeed, *Salmonella* species are thought to be the main factor contributing to diarrheal disease-associated deaths [1]. According to the World Health Organization, *Salmonella* accounts for approximately 9% of diarrheal illnesses and it costs 2.7 billion US dollars to care for these patients in the United States per year [2,3]. Especially, since food production is evolving to reflect demand for raw and lightly cooked foods, foodborne pathogens including *Salmonella* have continued to spread all over the world [4,5].

To detect *Salmonella* at the early stage of infection, a sensitive and reliable method is crucial. Various methods have been developed for *Salmonella* detection using molecular tools such as direct polymerase chain reaction (PCR) and multiplex quantitative PCR [6]. A PCR-based assay is currently the most commonly used tool for molecular screening of *Salmonella* [7]. However, PCR methods require specialized equipment that must be optimized for the amplification of target genes from samples, along with the extraction and purification steps carried out by a well-trained individual. Several technical issues such as long reaction times (about 2 h) and the requirement for well-purified nucleic acid are drawbacks for clinical application in many real-world settings. The lack of hardware resources in the field constitutes a critical barrier to the detection of pathogens directly from clinical samples [8]. Taken together, the limitations of conventional PCR-based methods make these assays difficult to use for point-of-care testing (POCT). POCT is a new concept in the laboratory-medicine discipline that enables screening for pathogens at the site where the infection occurs without transporting the test materials to well-equipped laboratories; by obviating the need for transport, POCT would be helpful to reduce the time necessary for clinical decision-making [9,10]. Therefore, POCT requires fast reaction time and simple equipment.

Loop-mediated isothermal amplification (LAMP) technology, an isothermal nucleic acid amplification method developed by Notomi et al. [11], can amplify target nucleic acids at a consistent temperature without changing the temperature for PCR cycling. The LAMP assay has been proven to be an effective tool for POCT in the field to test for infectious diseases [12-14]. There are several advantages of the LAMP technology. First, due to isothermal amplification, LAMP does not need a thermal cycler; therefore, this technology is ideal for POCT in the field. Second, the reaction time is much shorter than that of PCR, which is advantageous for rapid testing of highly contagious infections and preventing the spread of infections at an early stage. Third, the detection sensitivity of LAMP is higher than that of conventional PCR due to the loop-mediated amplification strategy. Fourth, LAMP is relatively less sensitive to PCR inhibitors, which can contaminate samples from sources such as feces; therefore, LAMP would be advantageous for minimizing the sample preparation procedure. During the LAMP reaction, the inner primers anneal by Watson-Crick complementarity to a region within the target and a hybridized loop structure is generated by strand invasion, which allows more efficient amplification for the synthesis of large amounts of DNA in a short time [11].

Several studies have reported LAMP assays targeting *Salmonella* in food [15,16]. The invA gene has been the most frequent target for *Salmonella* detection LAMP assays [15]. In this study, we aimed to develop a real-time LAMP assay targeting the *hilA* gene for universal detection of *Salmonella* species. We also validated the LAMP assay with seven clinically important *Salmonella* pathogens.

## Methods

### Bacterial strains

In this study, seven major *Salmonella* species were used. Six species (*Salmonella* Typhi, *Salmonella* Typhimurium, *Salmonella enteritidis*, *Salmonella* Paratyphi A, *Salmonella* Paratyphi B, and *Salmonella* Infantis) were collected from the Korea National Institute of Health (KNIH), Republic of Korea. *Salmonella enterica* was collected from Chungbuk Veterinary Service Laboratory, Chungju, Republic of Korea. For the specificity test, we used 11 non-*Salmonella* pathogens: seven Gram-negative and four Gram-positive bacteria (Table 1).

### DNA extraction and template DNA preparation

All *Salmonella* strains (n=7) were cultivated for 24 h at 37℃ on sheep blood agar (Bandio, Pocheon, Korea). Non-*Salmonella* strains (n=11) were cultivated for 36 hours at 37℃ on Difco Luria-Bertani agar (BD, Franklin Lakes, NJ, USA). DNA extraction was done via QlAamp DNA Mini kit (Qiagen, Germantown, MD, USA) according to the manufacturer’s instructions using the protocol. For Gram-negative bacteria, colonies on the freshly cultured bacteria were added to the ATL buffer (180 μL) containing proteinase K (20 μL; 20 mg/mL) and suspension. The sample was then mixed by vortexing and incubated at 56℃ until the bacteria were completely lysed. Next, 200 μL of lysis buffer was added to each sample and incubated at 70℃ for 10 min. Then, vortexing for 15 seconds was performed right after adding 200 μL of ethanol. The mixture was transferred to the QlAamp Mini Spin column and centrifuge at 6,000 ×g for 1 min. The genomic DNA in the spin column was washed using 500 μL of AW1 buffer and centrifuged at 6,000 ×g for 1 min. Next, 500 μL of AW2 buffer was added and centrifuged at 20,000 ×g for 3 min. For the elution process, 100 μL of distilled water was added and centrifuged at 6,000 ×g. The genomic DNA was measured using Qubit 3.0 Fluorometer (Thermo Fisher Scientific, Waltham, MA, USA) and the DNA was stored at ‒20℃ for further study. Gram-positive bacteria were added to 180 μL of lysis buffer containing proteinase K (20 μL; 20 mg/mL) and incubated for 30 min at 37℃ instead of using the ATL buffer. The other steps were the same as in the protocol for Gram-negative bacteria.

### Primer design for the *Salmonella* LAMP assay

**Fig. 1.**
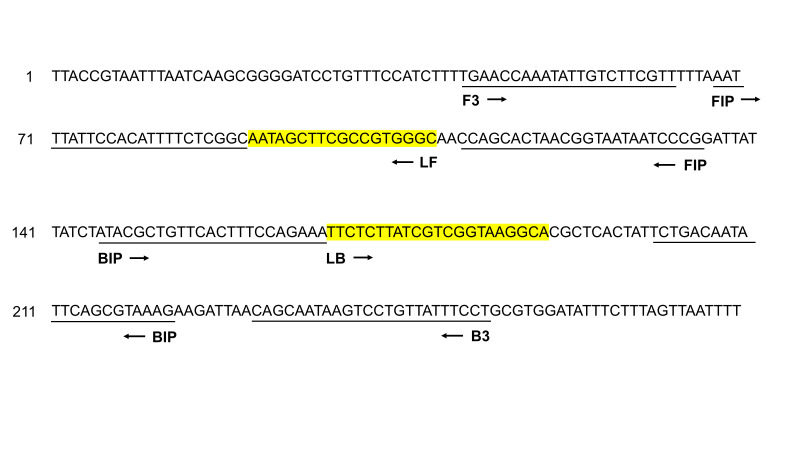
Primer design for the *Salmonella* loop-mediated isothermal amplification assay. The positions of six primers (F3, B3, FIP, BIP, LB, and LF) were aligned on the partial nucleotide sequence of the *hilA* gene (GeneBank accession No. CP075108). The forward/backward outer primers (F3/B3) and forward/backward inner primers (FIP/BIP) are underlined. The forward/backward loop primers (LF/LB) are highlighted in yellow. Arrowheads indicate the direction of the primers.

Primer Explorer V4 (http://primerexplorer.jp/elamp4.0.0/index.html) was used to design *Salmonella* species LAMP primers. The three sets of primers included a forward outer primer (F3), a backward outer primer (B3), a forward inner primer (FIP), a backward inner primer (BIP), and two loop primers: a forward loop primer (LF) and a backward loop primer (LB). All sets of primers were then validated by the BLAST program. Of the three sets of LAMP primers, the set that demonstrated the best amplification performance was selected as the *Salmonella* LAMP primer set (Fig. 1).

### LAMP assay

The LAMP reaction was carried as described elsewhere with some modifications [17]. In brief, a 20 μL reaction mixture was prepared that contained 1 μL of target genomic DNA along with 1.6 μM each of the primers FIP and BIP, 0.2 μM each of F3 and B3, 0.4 μM of LF and LB. 4U of the Bst 2.0 DNA polymerase (New England Biolabs, Ipswich, MA, USA), 8 mM of MgSO_4_ (New England Biolabs), 1.5 mM of dNTPs (Thermo Fisher Scientific), 1× isothermal amplification buffer (New England Biolabs), and 1.25 M of N-methyl formamide (NMF) and isobutylamide (IBA). Furthermore, 2 μM of SYTO 9 (Thermo Fisher Scientific) was added to enhance fluorescence in the presence of DNA in the real-time assay. The amplification reaction was performed at 60℃ for 45 min and terminated by heating at 80℃ for 3 min using the CFX96 Touch Real-Time PCR Detection System (Bio-Rad Laboratories, Hercules, CA, USA). The results were shown as a graph on the monitor of real-time analysis software (BioRad CFX Manager, Bio-Rad Laboratories). The fluorescence curve was captured from the BioRad CFX Manager graph.

### Optimization of the LAMP reaction and evaluation of the detection sensitivity and specificity

To optimize the amplification conditions, the LAMP reaction was performed at different temperatures (60℃–65℃). To evaluate the detection sensitivity of the LAMP assays, we tested the detection limit using *S. enterica* DNA, which was 10-fold serially diluted from 1 ng to 1 ag and used in the LAMP reaction. To test for non-specific amplification of non-*Salmonella* pathogens, we conducted the LAMP assay with seven non-*Salmonella* Gram-negative and four Gram-positive bacteria.

## Results

### Design of LAMP primers

**Fig. 2.**
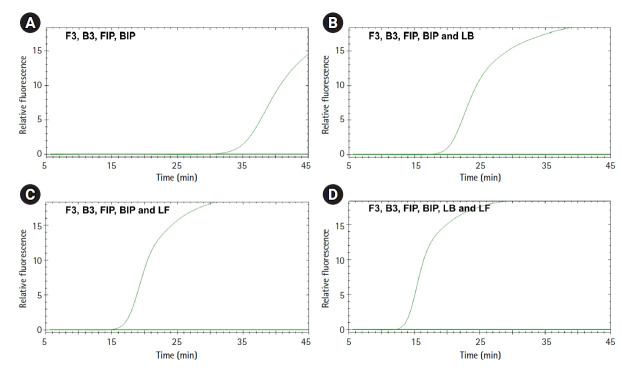
Optimization of the combinations of primers. Loop-mediated isothermal amplification (LAMP) reactions were performed with *Salmonella enterica* DNA with four different combinations of primers. (A) LAMP with two inner and two outer primers. (B) LAMP with the combination-A primers and one loop primer (LB). (C) LAMP with the combination-A primers and another loop primer (LF). (D) LAMP with all six primers. The x-axis represents the time needed for the LAMP reaction using a real-time PCR cycler; the y-axis represents the relative fluorescence signal. The flat amplification curve was found in the negative control.

Six LAMP primers were designed targeting the *hilA* gene as a universal marker of *Salmonella* species: two outer primers (F3 and B3), two inner primers (FIP and BIP), and two loop primers (LF and LB) (Fig. 1). Validation of the primer set was performed using *S. enterica* DNA under standard LAMP conditions (60℃ for 45 min) (Fig. 2). We performed the LAMP reaction with four different combinations of primers. Set 1 involved LAMP with two inner and two outer primers but without a loop primer, set 2 used the set 1 primers with one loop primer (LB), set 3 used the set 1 primers with another loop primer (LF), and set 4 used all six primers (Fig. 2). As expected, LAMP without the two loop primers showed the slowest amplification reaction. LAMP with one loop primer showed a faster amplification reaction than LAMP without any loop primer. LAMP with all six primers, including two loop primers, showed the fastest amplification reaction, in which a fluorescent signal appeared just 12 min after starting the reaction. None of the negative controls showed a positive signal during the reaction. Therefore, we decided to include all six primers in our *Salmonella* species LAMP system.

### Optimization of LAMP reaction conditions

**Fig. 3.**
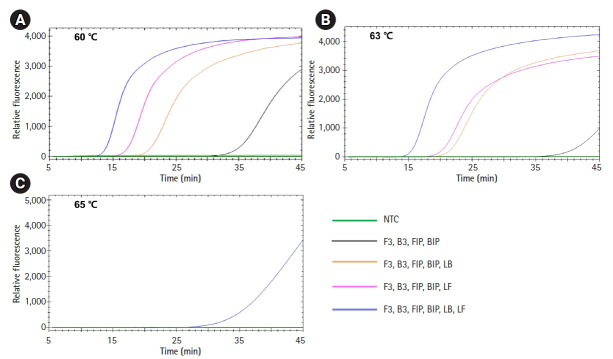
Optimization of loop-mediated isothermal amplification (LAMP) reaction conditions. LAMP reactions were performed with *Salmonella enterica* DNA under three different reaction conditions: 60℃ (A), 63℃ (B), and 65℃ (C). The combinations of primers were the same as shown in Fig. 2. NTC, negative control.

To determine the best LAMP conditions, we compared three different reaction conditions: 60℃, 63℃, and 65℃ for 45 min (Fig. 3). Consistent with the results in Fig. 2, LAMP with all six primers including two loop primers showed the fastest amplification reaction regardless of reaction temperature. Regarding the reaction conditions, a reaction temperature of 60℃ showed the best amplification performance. Integrating these, we set the conditions for our *Salmonella* LAMP assay as using all six primers at 60℃ for 45 min.

### Detection sensitivity of the LAMP assay

**Fig. 4.**
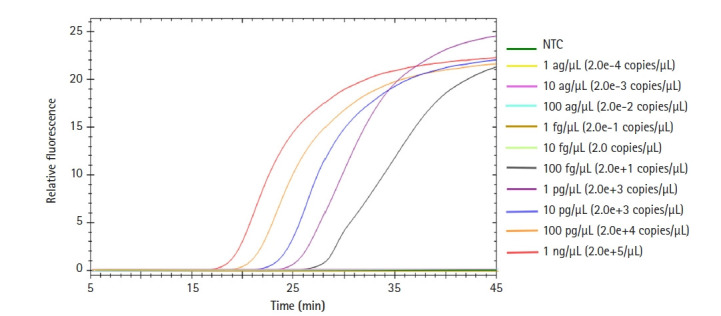
Sensitivity of the *Salmonella* species loop-mediated isothermal amplification (LAMP) assay. The template DNA (*Salmonella enterica* DNA) was 10-fold serially diluted (1 ng/μL to 1 ag/μL) and tested with our *Salmonella* LAMP assay. NTC, negative control.

To test the detection sensitivity of our *Salmonella* LAMP assay, we observed the limit of detection (LOD) of this assay. *Salmonella enterica* DNA was 10-fold serially diluted from 1 ng to 1 ag and used in the LAMP reaction (Fig. 4). The LOD of the *Salmonella* LAMP assay was 100 fg which corresponds to 20 copies of *Salmonella* DNA. The time for the fluorescent signal to appear from 1 ng of template DNA was less than 20 min. Even in the case of 1 pg of template DNA, the amplification signal appeared within 25 min.

### Validating the universal detection of *Salmonella* species and specificity

**Fig. 5.**
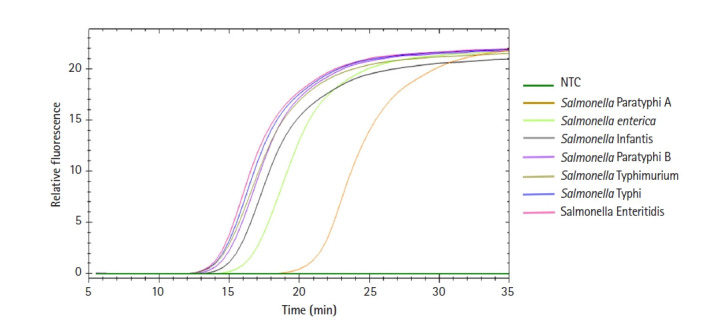
Universal detection of *Salmonella* species using our loop-mediated isothermal amplification (LAMP) assay. DNA extracted from seven clinically important *Salmonella* species (*Salmonella* Typhi, *Salmonella* Typhimurium, *Salmonella* Enteritidis, *Salmonella enterica*, *Salmonella* Paratyphi A, *Salmonella* Paratyphi B, *Salmonella* Infantis) was used in our *Salmonella* LAMP assay. NTC, negative control.

To validate whether our universal *Salmonella* LAMP assay can detect diverse *Salmonella* species, we prepared seven clinically important *Salmonella* species (*S.* Typhi, *S.* Typhimurium, *S.* Enteritidis, *S. enterica*, *S.* Paratyphi A, *S.* Paratyphi B, *S.* Infantis) and used them with this assay (Fig. 5). All seven *Salmonella* species showed specific amplification signals at around 15 min except *S.* Paratyphi A. In contrast, the negative control did not show any amplification signal. To verify the *Salmonella*-specific detection, we also conducted our *Salmonella* LAMP assay with 11 non-*Salmonella* pathogens (seven Gram-negative and four Gram-positive bacteria) (Table 1). None of the non-*Salmonella* pathogens showed any amplification signal with our *Salmonella* LAMP assay (data not shown).

## Discussion

In this study, to develop a real-time LAMP assay for the rapid and sensitive detection of *Salmonella* species, we designed a LAMP primer set targeting the *hilA* gene. The *hilA* gene, a member of the transcriptional regulator genes encoded in the *Salmonella* pathogenicity island 1 (SPI1), plays an important role in the pathogenesis of *Salmonella* infection by activating the expression of SPI1 [18,19]. SPI1 is one of the key virulence factors for *Salmonella* infection and invasion. The *hilA* gene is known to be *Salmonella* species-specific and absent in other Gram-negative bacteria [20]. Therefore, this gene has been targeted for detecting *Salmonella* infection by PCR [21]. However, no study has yet developing a LAMP assay targeting the *hilA* gene for the detection of *Salmonella* species.

In the LAMP assay, a high level of amplification efficiency can be achieved under isothermal conditions due to strand displacement by the Bst DNA polymerase enzyme [11]. However, the possibility of non-specific amplification is also high due to the high level of amplification. To minimize this possibility, we added NMF and IBA as described previously [17]. In addition, we optimized the LAMP reaction conditions to facilitate the efficient and reliable detection of *Salmonella* species.

When we checked the detection sensitivity of our *Salmonella* LAMP assay using *S. enterica* DNA, the LOD was 100 fg, which corresponds to just 20 copies of *Salmonella* DNA. In the previous PCR assay targeting the *hilA* gene using *S. typhimurium* DNA, the LOD was 100 pg [21]. Therefore, although the *Salmonella* strains used for the sensitivity test were different, our *Salmonella* LAMP assay is approximately 1,000 times more sensitive than the PCR-based assay to detect *Salmonella* species. Of note, when we tested whether our universal *Salmonella* LAMP assay can detect diverse *Salmonella* species, all seven *Salmonella* species (including *S.* Typhimurium) showed consistent detection performance, providing further support that our *Salmonella* LAMP assay is much more sensitive than the PCR assay. This result also suggests that this *Salmonella* LAMP assay is universally applicable for the detection of diverse clinically important *Salmonella* species.

When we tested the assay using 11 non-*Salmonella* pathogens, none of them showed any amplification signals with our *Salmonella* LAMP assay, suggesting high *Salmonella* species specificity of our LAMP assay. According to the BLAST (https://blast.ncbi.nlm.nih.gov/Blast.cgi) analysis, none of the 11 non-*Salmonella* pathogens showed similar sequences to the *hilA* gene (data not shown). Of particular note, a previous study based on the conventional PCR method targeting the *hilA* gene took approximately 1 hour and 45 minutes [21]. However, all *Salmonella* species tested in this study showed specific amplification signals at around 15 minutes except S. Paratyphi A, demonstrating that our *Salmonella* LAMP assay would be ideal for POCT.

One of the fundamental limitations of conventional PCR is that PCR is easily inhibited by substances in the sample. LAMP technology can overcome this drawback of PCR, as LAMP is relatively less sensitive to PCR inhibitors, which can contaminate samples from sources such as feces. Therefore, LAMP would be advantageous to minimize the sample preparation procedure.

In conclusion, our *Salmonella* LAMP assay targeting the *hilA* gene demonstrated a high level of sensitivity and specificity. Considering the higher sensitivity of our assay than conventional PCR, its rapid reaction time, and its lower sensitivity to potential PCR inhibitors, our assay could be a useful POCT tool for *Salmonella* species.

## Figures and Tables

**Table 1.** Strains used for testing the *Salmonella* LAMP assay based on the *hilA* gene

	Species	Strain No.
*Salmonella* strains		
1	*Salmonella* Typhi	NCCP 10820
2	*Salmonella* Typhimurium	NCCP 16207
3	*Salmonella* Enteritidis	NCCP 14547
4	*Salmonella* enterica	CVSL 0029
5	*Salmonella* Paratyphi A	NCCP 14759
6	*Salmonella* Paratyphi B	NCCP 12204
7	*Salmonella* Infantis	NCCP 12233
Non-*Salmonella* strains		
1	*Pseudomonas aeruginosa*	NCCP 14781
2	*Klebsiella pneumoniae*	NCCP 14764
3	*Enterobacter aerogenes*	NCCP 14761
4	*Escherichia coli*	NCCP 14538
5	*Acinetobacter baumannii*	NCCP 14782
6	*Shigella flexneri*	NCCP 14744
7	*Shigella sonnei*	NCCP 14773
8	*Streptococcus pneumoniae*	NCCP 15898
9	*Streptococcus pyogenes*	NCCP 14783
10	*Staphylococcus epidermidis*	NCCP 14768
11	*Streptococcus salivarius*	NCCP 16179

LAMP, loop-mediated isothermal amplification.
